# Correlation Analysis between Nerve Fiber Layer Thickness and Peripapillary Vessel Density and Influencing Factors of Peripapillary Vessel Density in Preclinical Diabetic Retinopathy

**DOI:** 10.1155/2020/2758547

**Published:** 2020-12-15

**Authors:** Donghui Li, Qichang Wang

**Affiliations:** ^1^Aier School of Ophthalmology, Central South University, Changsha 410000, China; ^2^Changsha Xiangjiang Aier Eye Hospital, Changsha 410000, China

## Abstract

**Purpose:**

To observe the changes of the retinal nerve fiber layer (RNFL) thickness and the optic disc vessel density (VD) in preclinical diabetic retinopathy (DR) and the relationship between RNFL changes and VD, as well as to investigate the influencing factors on peripapillary vessel density.

**Methods:**

This was a cross-sectional study. Thirty-four eyes of 34 type 2 diabetes mellitus (T2DM) patients diagnosed with preclinical diabetic retinopathy (DR) were included in our study, with twenty-three eyes of 23 healthy subjects set up as normal controls. History of diabetes, hypertension, and dyslipidemia was recorded in detail. All participants underwent color fundus photography (CFP), RNFL around the optic disc, and OCT angiography (OCTA) over the optic disc. The 4.5 mm × 4.5 mm Angio Disc scan mode was performed with all participants by using the OCTA instrument. The relationship between changes of RNFL in the four quadrants (superior, inferior, temporal, and nasal) and VD changes was analyzed.

**Results:**

Vessel density was significantly lower in the superior (*t* = −2.27) and temporal (*t* = −2.02) peripapillary sectors of diabetic eyes compared to normal eyes (*P* < 0.05). The retinal nerve fiber layer (RNFL) was significantly thinner in the temporal quadrant (*P* < 0.001) of diabetic eyes compared to normal eyes. Pearson correlation coefficient analysis showed a significant positive correlation between vessel density and RNFL thickness in the peripapillary region in the temporal (*r* = 0.468, *P* < 0.01) and superior (*r* = 0.612, *P* < 0.01) sectors. Multiple linear regression analysis showed that glycated hemoglobin (HbA1c) (*β* = −1.50, *P* < 0.01) and the duration of diabetes (*β* = −0.33, *P*=0.03) were associated with peripapillary vessel density.

**Conclusions:**

Preclinical DR presented optic disc microcirculation changes. Temporal RNFL thinning is an early sign of retinal neurodegeneration and is associated with temporal peripapillary vessel density reduction. The duration of diabetes and HbA1c are risk factors for peripapillary vessel density reduction in patients with preclinical DR.

## 1. Introduction

Diabetic retinopathy (DR) was previously considered as a microvascular disease. The microvascular dropout of the macular area in preclinical and early-stage DR has been frequently reported in recent OCTA studies. And Liu et al. [1] found a significant decrease in vessel density of radial peripapillary capillaries (RPCs) in the peripapillary region in patients with DR, which was similar to the results of a recent publication by Vujosevic et al. [2]. However, more and more studies have now shown that not only microvascular but also neural factors are associated with DR [3–5]. Furthermore, retinal neurodegeneration has been found to play a significant role in the pathogenesis of DR [6], as it is associated with decreased retinal nerve fiber layer (RNFL) thickness [7, 8]. Retinal neurodegeneration is described as a consequence of neuron apoptosis, reactive gliosis, glutamate excitotoxicity, decrease in neuroprotective factors, and impairment of the neurovascular coupling. RPCs tend to pursue relatively long, straight paths, which are parallel to the retinal ganglion cell axons. RPCs might be derived from the arterioles in the ganglion cell layer, and they arched up steeply to supply the superficial RNFL around the ONH [9]. Recent study [10] has demonstrated that microvascular alterations in the optic nerve head may occur earlier than the peripapillary RNFL defect in the course of DR.

Optical coherence tomography angiography (OCTA) has been considered as a useful tool to noninvasively investigate retinal microvascular changes and directly quantify the vessel density of the optic disc [11]. This study aimed to observe the changes of the retinal nerve fiber layer (RNFL) thickness and optic disc vessel density (VD) in preclinical diabetic retinopathy (DR) and the relationship between RNFL changes and VD, as well as to investigate the influencing factors on peripapillary vessel density.

## 2. Methods

### 2.1. Study Design and Participants

This was a cross-sectional study. The study was approved by the Ethics Committee of Changsha Xiangjiang Aier Eye Hospital (approval number: CXAECKY2018-01) and was conducted according to the Declaration of Helsinki. Thirty-four preclinical DR eyes of 34 type 2 diabetes mellitus patients and 23 eyes of 23 healthy controls were included. The participants were recruited at Xiangjiang Aier Eye Hospital in Changsha, China, from January to September 2019. All participants were informed of the aims and procedures of the study and signed informed consent forms. Data on the participants' age, gender, blood pressure, course of diabetes, glycated hemoglobin, triglyceride, total cholesterol, high-density lipoprotein, and low-density lipoprotein levels were recorded.

The inclusion criteria were as follows: (1) for the preclinical DR group, clinical diagnosis of type 2 diabetes; for the control group, no history of diabetes and high blood sugar. (2) No microaneurysm, bleeding points, or exudation in color fundus photography and fundoscopy after mydriasis induction. (3) Lens power spherical equivalent less than −6.00D. (4) OCTA image signal intensity ≥6. The exclusion criteria were as follows: (1) diabetic retinopathy in one eye. (2) History of eye trauma and surgery. (3) Intraocular pressure ≥21 mmHg and history of glaucoma. (4) Systolic pressure ≥140 mmHg or (and) diastolic pressure ≥90 mmHg. (5) Uveitis, retinal vein occlusion, age-related macular degeneration, or other retinal diseases.

### 2.2. Ocular Examination

Each patient underwent a series of ocular examinations that included a biomicroscopy examination of the fundus, OCTA, color fundus photography, and intraocular pressure measurement using a noncontact tonometer. Preclinical DR was confirmed based on the clinical assessment by retinal specialists and color fundus photography. The less seriously affected eye of each patient was chosen. If neither eye of a patient had retinopathy, the eye with the higher image signal intensity was chosen.

### 2.3. Optical Coherence Tomography Angiography Imaging and Image Processing

**Figure 1:**
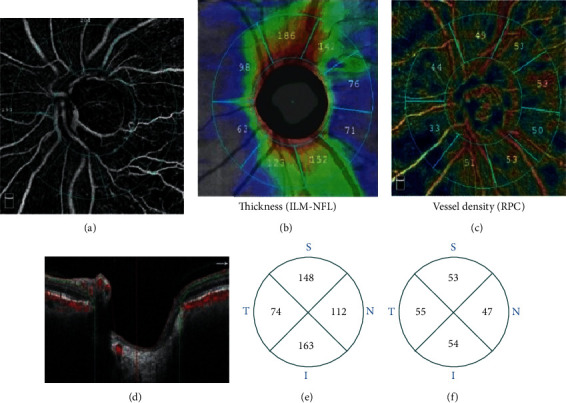
OCTA measures the parameters of the optic disc in the range of 4.5 mm × 4.5 mm. (a) Inside the optic disc: area surrounded by the inner blue circle; the peripapillary region: area between two blue rings. (b) RNFL thickness around the optic disc. (c) Peripapillary vessel density. (d) Peripapillary vessel measurement RPC layer range (ILM to NFL layer). (e) Peripapillary RNFL thickness four-quadrant zone. (f) Peripapillary vessel density four-quadrant zone.

OCTA imaging of the optic disc was performed using the AngioVue OCTA system (Optovue, Fremont, CA, USA). A 4.5 × 4 .5 mm rectangle scan centered on the optic nerve head was performed. The newly developed, built-in AngioAnalytics software was used to measure vessel density and RNFL thickness. Vessel densities for the whole 4.5 × 4.5 mm scan area (whole image), optic disc area (inside disc), and peripapillary region (a 0.75 mm wide annular area extending outward from the optic disc boundary) (Figure 1(a)), as well as average RNFL thickness for the entire peripapillary area and each sector, were generated by using automated software algorithm. The peripapillary region was divided into different sections; RPC density in these sections (Figure 1(c)) and peripapillary RNFL thicknesses (Figure 1(b)) were automatically calculated by software. The peripapillary vessels were analyzed in superficial retinal layers from the RPC segment that extends from the inner limiting membrane to the nerve fiber layer (Figure 1(d)). Peripapillary vessel density was defined as the percentage of the area occupied by vessels in the peripapillary region.

### 2.4. Statistical Analysis

SPSS software (version 19.0) was used for statistical analysis. All data were tested for Shapiro–Wilk normality. The measurement data were expressed as mean ± standard deviation. The difference between the measurement indicators between the two groups was compared using two independent sample *t*-tests. Pearson correlation analysis was used to analyze the correlation between the average RNFL thickness and the blood flow density. In the NDR group, some systemic factors and peripheral blood flow density were analyzed by multiple regression. *P* < 0.05 was considered statistically significant.

## 3. Results

### 3.1. Patient Characteristics

All demographic data and general clinical characteristics are shown in Table 1.

### 3.2. Analysis of Vessel Density in the Peripapillary Region

In the preclinical DR group, the whole image vessel density of the optic disc was 54.68% ± 3.10%, the peripapillary vessel density was 56.39% ± 3.80%, and the vessel density inside the disc was 56.34% ± 5.47%. In the control group, the total vessel density of the optic disc was 56.54% ± 2.11%, the peripapillary vessel density was 59.29% ± 2.60%, and the vessel density inside the disc was 59.63% ± 5.58%. The preclinical group showed lower vessel density in the whole image (*t* = −2.51, *P*=0.015), inside the disc area (*t* = −2.20, *P*=0.03), and the peripapillary area (*t* = −3.18, *P* < 0.01) (Table 2).

### 3.3. Analysis of Nerve Fiber Layer Thickness in the Peripapillary Region

**Figure 2:**
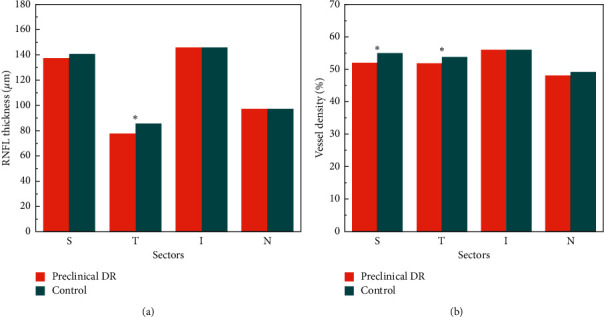
(a) RNFL thickness in each quadrant of the preclinical DR group and control group. (b) Vessel density in each quadrant of the preclinical DR group and control group.

The RNFL around the temporal disc was significantly thinner in the preclinical group than in the control group (*t* = −2.20, *P*=0.04). The vessel density in the superior and temporal sectors of the peripapillary area was significantly lower in the preclinical group than in the control group (*t* = −2.27, *P*=0.027 and *t* = −2.15, *P*=0.03) (Table 3 and Figure 2).

### 3.4. Correlations between Vessel Density and Retinal Nerve Fiber Layer Thickness

**Figure 3:**
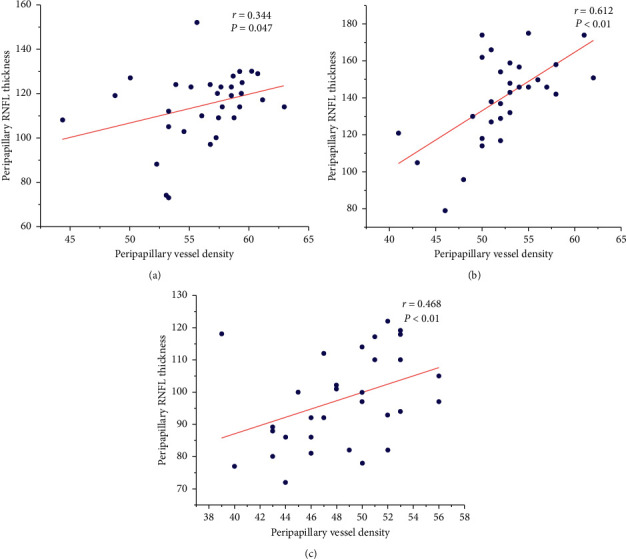
Scatter plots of RNFL thickness and peripapillary vessel density in the preclinical group. (a) Scatter plot of average RNFL thickness and vessel density. (b) Scatter plot of superior RNFL thickness and vessel density. (c) Scatter plot of temporal RNFL thickness and vessel density.

In the preclinical DR group, the average RNFL thickness positively correlated with peripapillary vessel density (*r* = 0.344, *P*=0.047). The RNFL thickness in the upper and temporal discs positively correlated with blood flow density in the respective areas (*r* = 0.612, *P* < 0.01 and *r* = 0.468, *P* < 0.01; Table 4 and Figure 3).

### 3.5. Multiple Regression Analysis of Peripapillary Vessel Density and Systemic Factors

**Figure 4:**
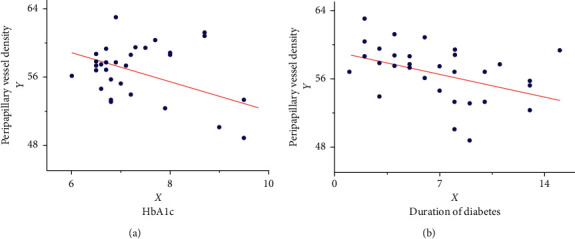
Scatter plots of peripapillary density and influencing factors.

Scatter plots of peripapillary vessel density and systemic factors show that the duration of diabetes and HbA1c have a negative linear relationship with peripapillary vessel density (Figure 4); triglycerides, cholesterol, high-density lipoprotein, and low-density lipoprotein have no obvious linear relationship with peripapillary density, so they are not included in the linear regression equation (Figure 4).

**Figure 5:**
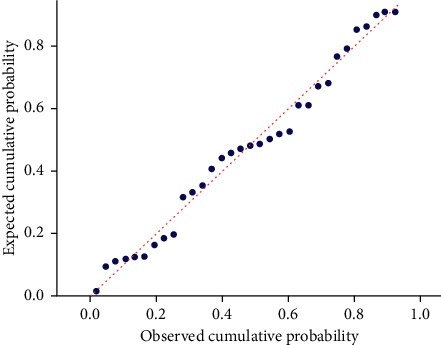
P-P plot graph.

**Figure 6:**
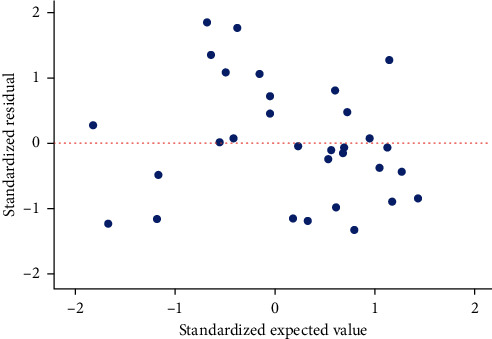
Plot for the standardized residual.

Using the SPSS stepwise regression analysis method, the model equation was established as *Y* = 69.91 − 0.33X_1_ − 1.50X_2_ (Table 5) (*X*_1_: diabetes duration; *X*_2_: HbA1c). The *F*-test results of the regression model showed that the linear relationship of the equation was significant (*F* = 7.75, *P* < 0.05). The Durbin–Watson test was used to determine whether the dependent variable values were independent of each other. The *P* value was not significant (*P*=1.72), indicating that there was no autocorrelation between the dependent variables and that the errors were independent of each other. The model's residual normal probability plot (P-P plot) shows that the points are basically scattered around the straight line, indicating that the residuals basically conform to the normal distribution (Figure 5). In the standardized residual scatter plot (Figure 6), most of the residual values are distributed in the band region of [−2, 2], and the model meets the condition of homogeneity of residual variance. The multiple linear regression model verifies that the expansion factors of the respective variables are between 1.023 and 1.024, which are all less than 10, and the multicollinearity is not significant.

## 4. Discussion

In normal human optic discs, the superficial blood supply of the retina comes from the central retinal artery, and the deep retinal blood supply originates from the posterior ciliary artery [12]. The optic disc RPC network is located inside the RNFL, parallel to the retinal ganglion cell axon, and supplies nutrients to the RNFL on the optic disc surface [13]. Due to its long and straight structural features, it lacks an anastomotic branch with the nerve fiber bundle, which makes it prone to pathological changes. More and more research has demonstrated the occurrence of neurovascular change in diabetic patients with no clinically visible lesions [10, 14]. With the advance of OCTA, the reduction of vessel density of RPCs in preclinical diabetes has been found. Our study shows that vessel density in the peripapillary region and inside the optic disc decreases in preclinical DR eyes, which was similar to the results of Cao et al. [10] who observed a reduction in vessel density of the RPC in the optic disc region in patients in preclinical DR. In addition, our study also revealed the reduction of vessel density in the superior and temporal quadrant in the peripapillary region, which differed from the results of Cao et al. [10] who considered that RPC density in all eight sectors in diabetic patients in preclinical DR was significantly lower than controls. The differences between our results and results of Cao et.al. may be due to the inconsistent method of the peripapillary region division.

Previous studies [15, 16] have demonstrated that RNFL thickness alteration was associated with RPC changes in early-onset glaucoma. These studies offered us further insights on the potential association between early loss of the RNFL in diabetic patients and RPC vessel density. Li et al. [17] found that the RNFL thickness of preclinical DR patients was decreased in the inferior and nasal quadrants compared with healthy subjects. However, our current study suggested different results in the preclinical DR group. We found that the RNFL thickness of the temporal disc in patients in preclinical DR was decreased. The reason may be due to the fact that the optic disc macular tract is on the temporal side of the optic disc, accounting for 1/4 of the optic nerve cross section, and it is sensitive to pathological damage. As shown in our study that there was a positive correlation between the peripapillary temporal RNFL and vessel density (*r* = 0.468, *P* < 0.01), we speculate that decreased RPC vessel density results in insufficient nutritional supply to the RNFL in the corresponding area, leading to a decrease in RNFL thickness. This is consistent with the results of Cao et al. [10] who used OCTA to study the optic disc area of diabetic patients and found that the optic disc vessel density of patients without DR was significantly lower than that of healthy individuals and that changes in the optic disc blood flow occurred earlier than the optic disc nerve fiber layer changes.

Diabetes duration and HbA1c have been shown to be high-risk factors for DR [18]. Our results showed that the duration of diabetes negatively correlated with peripapillary vessel density, which was consistent with Kohei's results [19]. The reason may be that the longer the course of diabetes is, the longer it is exposed to various risk factors, and pathological changes such as peripheral cell apoptosis, endothelial cell swelling, and thickening of the basement membrane progressively increase, and peripheral blood flow decreases significantly.

According to the level of HbA1c, Zhu [20] divided DR patients into three groups (group1: HbA1c < 7%, group2: HbA1c 7–9%, group3: HbA1c > 9%) Research on retinal vessel density shows that an increase in HbA1c in diabetic patients reduces vessel density. This may be due to a positive correlation between HbA1c levels and the erythrocyte aggregation rate. Erythrocyte aggregation leads to the formation of small arterial thrombi, resulting in an increase in blood flow resistance and a decrease in vessel density. The results of our study show that the HbA1c level in diabetic patients negatively correlates with peripapillary vessel density. This suggests that if HbA1c concentration is controlled, diabetic patients may maintain good optic disc blood flow. Therefore, the management of HbA1c may play an important role in the prevention of DR.

This study has some limitations. The main limitation is that we analyzed blood perfusion in an optic disc area of 4.5 × 4.5 mm. We did not include the vessel density of the macular area. The vessel density of the non-DR eye may change in the optic disc area and the macular area. Moreover, this study included only preclinical DR patients. We did not analyze optic disc vessel density in DR patients. Furthermore, there may be a relationship between the factors included in the multiple regression analysis, which requires further analysis. Although multiple regression analysis indicated that the duration of diabetes and the HbA1c and blood lipid levels are related to peripapillary vessel density reduction, the correlation was weak. One explanation may be that peripapillary vessel density may be affected by many other factors. Finally, the sample size of this study was small. Furthermore, studies with larger sample sizes are needed.

In conclusion, vessel density in the peripapillary region and inside the optic disc decreases in diabetic eyes, suggesting changes in the optic disc microcirculation in preclinical DR. Temporal RNFL thinning is an early sign of retinal neurodegeneration and is associated with temporal peripapillary vessel density reduction. The duration of diabetes and HbA1c are risk factors for peripapillary vessel density reduction in patients with preclinical DR. Early management and effective blood glucose control in diabetic patients may be beneficial for preventing RNFL thinning in the temporal disc.

## Figures and Tables

**Table 1:** Demographic and clinical characteristics of the study participants.

	Preclinical group	Control group	*t*	*P*
Age (years)	62.41 ± 9.28	63.04 ± 6.96	−0.28	0.78
Diabetic duration (years)	7.79 ± 4.14	—	—	—
Systolic pressure (mmHg)	136.35 ± 13.46	132.65 ± 15.33	0.96	0.34
Diastolic pressure (mmHg)	82.79 ± 7.51	80.22 ± 9.66	1.13	0.26
HbA1c (%)	7.42 ± 1.06	5.45 ± 0.70	7.82	＜0.01^*∗*^
TC (mmol/L)	4.66 ± 1.24	2.39 ± 1.38	6.45	＜0.01^*∗*^
TG (mmol/L)	1.72 ± 0.52	1.08 ± 0.42	4.93	＜0.01^*∗*^
HDL-c (mmol/L)	1.64 ± 0.40	1.37 ± 0.43	2.36	0.02
LDL-c (mmol/L)	2.73 ± 0.62	2.27 ± 0.94	2.06	0.047
LogMAR BCVA	0.10 ± 0.15	0.11 ± 0.10	−2.50	0.80
IOP (NCT) (mmHg)	14.02 ± 2.42	13.38 ± 1.60	1.06	0.30

HbA1c: glycosylated hemoglobin; TC: total cholesterol; TG: triglycerides; HDL-c: high-density lipoprotein; LDL-c: low-density lipoprotein; BCVA: best-corrected visual acuity; IOP: intraocular pressure; ^*∗*^ indicates *P* < 0.01, with statistics learn meaning.

**Table 2:** Vessel density in the preclinical group and control group.

	Preclinical group	Control group	*t*	*P*
Whole image (%)	54.68 ± 3.10	56.54 ± 2.11	−2.51	0.015^*∗*^
Inside disc (%)	56.34 ± 5.47	59.63 ± 5.580	−2.20	0.03^*∗*^
Peripapillary (%)	56.39 ± 3.80	59.29 ± 2.60	−3.18	＜0.01^*∗*^

^*∗*^ *P* < 0.05, with statistics learn meaning.

**Table 3:** RNFL thickness and vessel density in each sector.

	Preclinical DR group	Control group	*t*	*P*
Peripapillary RNFL thickness				
S (*μ*m)	140.97 ± 22.21	138.43 ± 13.20	0.54	0.59
T (*μ*m)	78.00 ± 8.30	86.35 ± 21.93	−2.02	0.04^*∗*^
I (*μ*m)	145.29 ± 22.95	146.00 ± 18.67	−0.12	0.90
N (*μ*m)	96.29 ± 17.30	96.70 ± 11.09	−0.10	0.92

Peripapillary vessel density				
S (%)	52.44 ± 4.29	54.74 ± 2.73	−2.27	0.027^*∗*^
T (%)	52.96 ± 3.82	54.35 ± 6.10	−2.15	0.03^*∗*^
I (%)	55.71 ± 13.63	55.91 ± 4.86	−0.07	0.95
N (%)	48.38 ± 4.21	49.30 ± 3.60	−0.86	0.40

S: superior; T: temporal; I: inferior; N: nasal.

**Table 4:** Correlations between vessel density and RNFL.

	Average	S	T	I	N
*r*	0.344	0.612	0.468	0.056	−0.103
*P*	0.047	＜0.01	＜0.01	0.70	0.56

^*∗*^ *P* < 0.05, with statistics learn meaning.

**Table 5:** Multiple regression analysis of peripheral blood flow density factors and systemic factors.

Variate	*β*	*t*	*P*	95% CI
Constant term	69.91	17.67	＜0.01	[61.84, 77.98]
Diabetes duration (year)	−0.33	−2.27	0.03	[−0.62, −0.03]
HbA1c (%)	−1.50	−2.83	＜0.01	[−2.58, −0.42]

## Data Availability

The data used to support the findings of this study are included within the article.

## References

[1] Liu L., Wang Y., Liu H. X., Gao J. (2019). Peripapillary region perfusion and retinal nerve fiber layer thickness abnormalities in diabetic retinopathy assessed by OCT angiography. *Translational Vision Science & Technology*.

[2] Vujosevic S., Muraca A., Gatti V. (2018). Peripapillary microvascular and neural changes in diabetes mellitus: an OCT-angiography study. *Investigative Opthalmology & Visual Science*.

[3] Sohn E. H., Van Dijk H. W., Jiao C. (2016). Retinal neurodegeneration may precede microvascular changes characteristic of diabetic retinopathy in diabetes mellitus. *Proceedings of the National Academy of Sciences*.

[4] Frydkjaer-Olsen U., Soegaard Hansen R., Pedersen K., Peto T., Grauslund J. (2015). Retinal vascular fractals correlate with early neurodegeneration in patients with type 2 diabetes mellitus. *Investigative Opthalmology & Visual Science*.

[5] Garcia-Martin E., Cipres M., Melchor I. (2019). Neurodegeneration in patients with type 2 diabetes mellitus without diabetic retinopathy. *Journal Ophthalmol*.

[6] Iwona B.-S. (2016). Growth factors in the pathogenesis of retinal neurodegeneration in diabetes mellitus. *Current Neuropharmacology*.

[7] Shahidi A. M., Sampson G. P., Pritchard N. (2012). Retinal nerve fibre layer thinning associated with diabetic peripheral neuropathy. *Diabetic Medicine*.

[8] Chen X., Nie C., Gong Y. (2015). Peripapillary retinal nerve fiber layer changes in preclinical diabetic retinopathy: a meta-analysis. *PLoS One*.

[9] Yu P. K., Cringle S. J., Yu D.-Y. (2014). Correlation between the radial peripapillary capillaries and the retinal nerve fibre layer in the normal human retina. *Experimental Eye Research*.

[10] Cao D., Yang D., Yu H. (2019). Optic nerve head perfusion changes preceding peripapillary retinal nerve fibre layer thinning in preclinical diabetic retinopathy. *Clinical & Experimental Ophthalmology*.

[11] Mo S., Phillips E., Krawitz B. D. (2017). Visualization of radial peripapillary capillaries using optical coherence tomography angiography: the effect of image averaging. *PLoS One*.

[12] Hayreh S. S. (1969). Blood supply of the optic nerve head and its role in optic atrophy, glaucoma, and oedema of the optic disc. *British Journal of Ophthalmology*.

[13] Scoles D., Gray D. C., Hunter J. J. (2009). In-vivo imaging of retinal nerve fiber layer vasculature: imaging histology comparison. *BMC Ophthalmology*.

[14] Dimitrova G., Chihara E., Takahashi H., Amano H., Okazaki K. (2017). Quantitative retinal optical coherence tomography angiography in patients with diabetes without diabetic retinopathy. *Investigative Opthalmology & Visual Science*.

[15] Mammo Z., Heisler M., Balaratnasingam C. (2016). Quantitative optical coherence tomography angiography of radial peripapillary capillaries in glaucoma, glaucoma suspect, and normal eyes. *American Journal of Ophthalmology*.

[16] Mansoori T., Sivaswamy J., Gamalapati J. S., Balakrishna N. (2017). Radial peripapillary capillary density measurement using optical coherence tomography angiography in early glaucoma. *Journal of Glaucoma*.

[17] Li Z., Wen X., Zeng P. (2019). Do microvascular changes occur preceding neural impairment in early-stage diabetic retinopathy? evidence based on the optic nerve head using optical coherence tomography angiography. *Acta Diabetologica*.

[18] Du Z. D., Hu L. T., Zhao G. Q. (2011). Epidemiological characteristics and risk factors of diabetic retinopathy in type 2 diabetes mellitus in Shandong Peninsula of China. *International Journal of Ophthalmology*.

[19] Ichinohasama K., Kunikata H., Ito A. (2019). The relationship between carotid intima-media thickness and ocular circulation in type-2 diabetes. *Journal of Ophthalmology*.

[20] Zhu (2019). Quantitative analysis of the effect of HbA1c level on macular microcirculation in patients with type 2 diabetes mellitus. *Chinese Journal of Ocular Fundus Diseases*.

